# The effectiveness and safety of continuous and repeated treatment of atosiban in twin pregnancy with threatened preterm labor: A propensity score-matched study

**DOI:** 10.1371/journal.pone.0328008

**Published:** 2025-07-09

**Authors:** Hao Zhu, Weirong Gu, Bian Wang, Rong Hu

**Affiliations:** 1 Department of Obstetrics, Obstetrics & Gynecology Hospital of Fudan University, Shanghai Key Lab of Reproduction and Development, Shanghai Key Lab of Female Reproductive Endocrine Related Diseases, Shanghai, China; 2 Department of Assisted Reproduction, Shanghai Ninth People’s Hospital Affiliated with JiaoTong University School of Medicine, Shanghai, China; University of the Witwatersrand, SOUTH AFRICA

## Abstract

**Purpose:**

This retrospective cohort study aimed to evaluate the effects and adverse outcomes of continuous and repeated treatment with atosiban in twin pregnancies with threatened preterm labor.

**Patients and methods:**

The study was conducted at the Department of Obstetrics, Obstetrics and Gynecology, Hospital of Fudan University and included 90 twin pregnancies diagnosed with threatened preterm labor between January 2018 and December 2022. The pregnancies received atosiban tocolytic treatment. The data was divided into two groups based on whether continuous and repeated treatment was administered. Delivery outcomes, as well as maternal and neonatal complications, were analyzed. Propensity score matching (PSM) was employed to create comparable groups.

**Results:**

Out of the 90 women, 34 received continuous and repeated treatment, while 56 did not. After PSM, 33 women who received continuous and repeated treatment were matched with 33 women who did not. The continuous and repeated treatment group showed a significant prolongation of pregnancy (p = 0.001) with a hazard ratio of 0.58 (95%CI, 0.35–0.96), and a later gestational age (p = 0.042). These patients also had a more complete cycle of antenatal steroids (p = 0.002) and a lower proportion of cases requiring operative delivery due to fetal distress (OR=0.14 [0.03–0.75], p = 0.004). Additionally, neonates in the continuous and repeated treatment group had a lower rate of admission to the neonatal intensive care unit (NICU) (OR=0.41 [0.19–0.89], p = 0.023).

**Conclusion:**

This propensity score-matched study suggests that continuous and repeated treatment with atosiban may effectively prolong pregnancy in cases of twin pregnancies with threatened preterm labor. Continuous and repeated treatment with atosiban was associated with lower fetal distress and lower NICU admission but no effect in adverse neonatal outcomes as well as the incidence of chorioamnionitis.

## Introduction

With the continuous advancement of assisted reproductive technology, the global incidence of twin pregnancies has significantly increased [1]. In the United States, the rate of twin pregnancies in 2021 was reported to be 3.15% [2]. Among these pregnancies, 62.05% resulted in preterm births (<37 weeks), with 20.28% classified as early preterm births (<34 weeks). These rates were 7.5–10 times higher compared to singleton pregnancies [2]. In China, preterm birth, including both singleton and multiple pregnancies, is defined as delivery occurring between 28 + 0 weeks and less than 36 + 6 weeks of gestation. Accurate data on preterm birth rates in twin pregnancies in China is limited, but a report from 2019 indicated a twin pregnancy rate of 3.69% and a preterm birth rate of 60.78% [3].

Preterm birth poses a significant risk to both neonatal and infant health in twin pregnancies. Twins have a 5-fold higher risk of early neonatal and infant death related to prematurity [4]. Preterm newborns are more susceptible to short-term complications, including respiratory distress syndrome (RDS), intraventricular hemorrhage, necrotizing enterocolitis, and sepsis. They are also at risk of long-term morbidities, such as chronic lung disease and neurological disabilities [5]. Tocolysis, through the use of medications to inhibit uterine contractions, can potentially prolong pregnancy and allow for further in utero maturation and interventions that may improve infant outcomes [6].

In twin pregnancies, there are greater maternal hemodynamic changes compared to singleton pregnancies, which increases the risk of maternal cardiopulmonary complications, such as pulmonary edema [7,8]. Additionally, twin pregnancies are more prone to maternal metabolic complications [9,10]. Therefore, when managing threatened preterm labor in twin pregnancies, the use of tocolytics should be carefully considered. Since data on the prevention of preterm labor in twin pregnancies is lacking, management strategies used for singleton pregnancies are often applied in clinical practice. Commonly used tocolytics include beta-agonists, calcium channel blockers, prostaglandin inhibitors, and oxytocin receptor blockers. While the first three drugs are relatively affordable, they are associated with a risk of maternal side effects, such as maternal tachycardia, palpitations, hypotension, pulmonary edema, and metabolic effects, including hyperglycemia [11–14]. Furthermore, prostaglandin inhibitors pose fetal risks of ductus arteriosus constriction and oligohydramnios [15]. Atosiban, a dual oxytocin/vasopressin V1A antagonist approved for clinical use in women at risk of imminent preterm labor, is an alternative option [16]. It works by competing with oxytocin for receptor binding in the myometrium, decidua, and fetal membranes, thereby reducing oxytocin’s efficacy and calcium ion levels in muscle cells, which helps to inhibit uterine contractions, and there are no absolute contraindications to its use [17,18].

Although guidelines have traditionally recommended a 48-hour tocolytic therapy for antenatal corticosteroids to promote fetal lung maturation and facilitate transportation to a perinatal center, the benefits of prolonging gestational age have been recognized. Neonatal morbidities and mortalities are dependent on the gestational age at delivery [19]. Continuous and repeated treatment with tocolytics is possible and promising, particularly for pregnancies that are far from term. Moreover, studies have gradually shifted their focus towards continuous treatment with tocolytics and maternal safety during medication [20]. It has been found that the use of tocolytics benefits pregnancy prolongation after 34 weeks of gestation, allowing more time for intrauterine transfer [21].

There is limited research available on the use of atosiban in multiple pregnancies and the recommendations for preterm labor management. The efficiency and safety of continuous and repeated treatment with atosiban in twin pregnancies remain unclear. Therefore, the aim of this study was to assess the effects and maternal safety of continuous and repeated treatment with atosiban in twin pregnancies, while monitoring maternal and neonatal morbidities. The findings of this study may provide valuable insights for clinical treatment strategies.

## Materials and methods

### Study design and patients

This retrospective cohort study included women with twin pregnancies who were admitted to the Obstetrics and Gynecology Hospital of Fudan University in Shanghai, China, between January 2018 and December 2022, for tocolytic therapy using atosiban. The respective neonatal records were also reviewed retrospectively.

Inclusion criteria were patients with twin pregnancies, who received tocolytic therapy using atosiban. Patients who had congenital fetal anomalies were excluded from the study. The collected data included maternal age, gravidity, parity, maternal comorbidities (such as hypertensive disease in pregnancy, gestational diabetes mellitus, in vitro fertilization and embryo transfer [IVF-ET] and cervical problems, including suspected cervical incompetence and past cervical surgery), history of preterm labor, chorionicity of the twin pregnancy, gestational age at the start of atosiban treatment, latency period, gestational age at birth, administration of antenatal steroids, delivery mode, neonatal bodyweight, Apgar scores at 1, 5, and 10 minutes, admission to the neonatal intensive care unit (NICU), and duration of hospitalization. Adverse outcomes were also recorded, including maternal antenatal outcomes such as chorioamnionitis and sepsis, and postnatal outcomes such as postpartum hemorrhage, postpartum hypoxemia, puerperal infection, and venous thromboembolism (VTE). The neonatal adverse outcomes consisted of respiratory distress syndrome (RDS), acidosis (arterial blood gas pH < 7.3), wet lung, sepsis, hypoglycemia, feeding intolerance, and neonatal death.

### Atosiban treatment

The administration of atosiban followed the prescribed directions and strictly adhered to the indications and contraindications. Experienced obstetricians specialized in managing premature labor initiated and monitored the treatment. In our hospital, pregnant women considered to have a threatened preterm labor undergo tocolytic therapy based on the following physical examination criteria: (1) regular uterine contractions lasting at least 30 seconds with a frequency of every 5–6 minutes, and (2) cervical dilation (less than 3 cm) with more than 50% effacement. A complete therapy course lasts 48 hours and consists of three consecutive stages: an initial bolus dose (6.75 mg) of atosiban solution for injection (7.5 mg/ml), followed immediately by a high-dose continuous infusion (loading infusion of 300 μg/min) of atosiban 7.5 mg/ml concentrate for infusion, and subsequently, a lower-dose infusion (subsequent infusion of 100 μg/min) of atosiban 7.5 mg/ml concentrate for infusion for up to 45 hours. For repeat treatments, experienced obstetricians evaluate the maternal and fetal condition comprehensively and thoroughly. If repeat treatment is deemed necessary, it involves a bolus injection of atosiban 7.5 mg/ml solution for injection followed by an infusion of atosiban 7.5 mg/ml concentrate for infusion. Non-repeated treatment is defined as only one complete therapy course or less than 48 hours of atosiban infusion, following the protocol. Continuous and repeated treatment is defined as more than one complete therapy course (over 48 hours of atosiban infusion) in the present study. There are two occasions for the administration of atosiban: (1) the withdrawal period after one cycle of treatment, where the reuse of atosiban starts with a bolus dose, followed by a high-dose infusion for 3 hours and a low-dose maintenance infusion, and (2) the continuation period after one cycle of treatment, which involves a continued low-dose infusion of 100 μg/min. Maternal and fetal conditions are closely monitored during the medication period. If obstetric risks arise (such as fetal distress, chorioamnionitis, or cervical dilation exceeding 4 cm), the medication will be discontinued.

### Corticosteroids for fetal lung maturity

Patients before term were administered 5 mg of dexamethasone intramuscularly every 12 hours for a duration of 2 days, following our hospital’s clinical protocol. In cases where applicable, women with a gestational age of less than 34 weeks, and whose previous course of antenatal corticosteroids was administered more than 14 days ago, may be considered for a single repeat course of antenatal corticosteroids [22].

### Magnesium sulphate for fetal neuroprotection

For pregnant women with a gestational age below 34 weeks, intravenous administration of 4 g magnesium sulphate over a 30-minute period before delivery was carried out in accordance with our hospital’s clinical protocol [23].

### Statistical analysis

Data analysis was performed using SPSS 25.0 (IBM Corp., Armonk, NY) and R software version 4.2.3. Continuous data were presented as mean and standard deviation or interquartile range, depending on the data distribution. Categorical variables were expressed as frequencies and percentages. In this study, post hoc randomization was applied, and a propensity score matching (PSM) approach at a 1:1 ratio was used to correct for estimation bias. PSM is a commonly employed research method to mitigate the influence of selection bias and covariate imbalance by using a linear combination of covariates and compressing the relevant factors into a single score. The propensity score was calculated using the treatment protocol type (continuous and repeated treatment or non-repeated treatment) as the dependent variable, and maternal factors [maternal age, gravidity, parity, hypertensive disease in pregnancy (yes or no), gestational diabetes mellitus (yes or no), cervical incompetence (yes or no), IVF-ET (yes or no), chorionicity (monochorionic diamniotic (MCDA) or dichorionic diamniotic (DCDA)), and gestational age at treatment] as predictors. Individuals with similar propensity scores were then compared between groups. Standardized mean difference (SMD) was used to assess covariate distribution balance between treatment groups. Chi-square tests, Mann-Whitney U-tests, or Student’s t-tests were performed, depending on data type, distribution, and sample size, to test hypotheses related to baseline characteristics and neonatal outcomes within the matched groups. Kaplan-Meier estimates and the Log-rank test were employed to evaluate the effect of atosiban treatment on gestational age prolongation. A P-value <0.05 was considered statistically significant.

### Ethics statement

Approval for this study was granted by the medical ethics committee of Obstetrics and Gynecology Hospital of Fudan University (2023−65). A waiver of informed consent was granted by the institutional review board due to the retrospective nature of the study.

## Results

### Baseline characteristics

**Fig 1:**
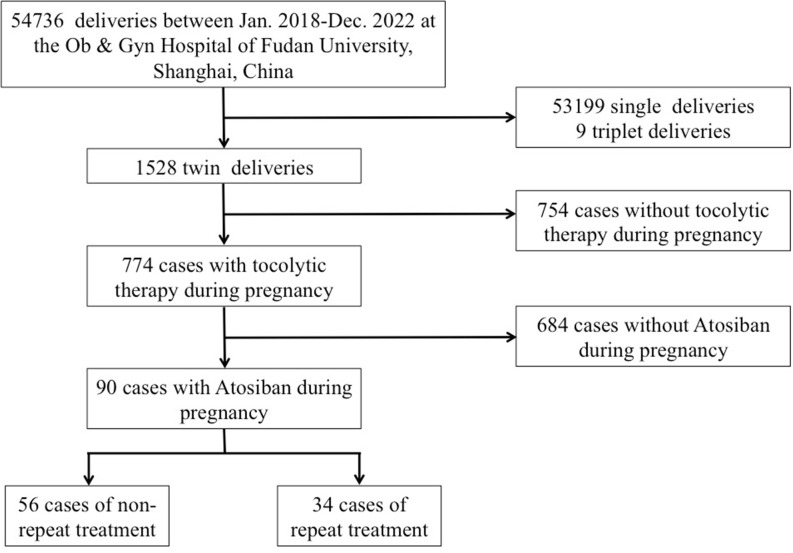
The flowchart that illustrates the process of patient inclusion in the study.

A total of 54,376 deliveries were identified, excluding 53,199 single deliveries and 9 triplet deliveries. Among the remaining 1,528 twin deliveries, 774 cases did not receive atosiban treatment and were subsequently excluded. Finally, 90 twin pregnancies that received atosiban treatment during pregnancy were included in the study (Fig 1). Among these, 34 women were in the continuous and repeated treatment group, while 56 women were in the non-repeated treatment group. Their ages ranged from 23 to 49 years, and they underwent atosiban treatment between 24 + 1 and 33 + 6 weeks of gestation. None of the women had a history of prior preterm labor. Table 1 presents a comparison of baseline characteristics between the two groups before and after propensity score matching. In the overall study group, there were no significant differences between groups in terms of maternal age, gravidity, history of hypertensive disease in pregnancy, gestational diabetes mellitus, cervical problems, IVF-ET, or chorionicity of twin pregnancy. However, differences were observed in parity (1.0 in the continuous and repeated treatment group vs. 1.3 in the non-repeated treatment group, p = 0.018) and gestational age at treatment (29.0 weeks in the continuous and repeated treatment group vs. 30.2 weeks in the non-repeated treatment group, p = 0.041) between the groups. After matching, no differences were observed for baseline variables.

**Table 1:** Comparison of baseline characteristics before and after matching.

	Before matching (n = 90)				After matching (n = 66)			
	Continuous and repeated treatment (n = 34)	Non-repeated treatment (n = 56)	SMD	*p* value	Continuous and repeated treatment (n = 33)	Non-repeated treatment (n = 33)	SMD	*p* value
Maternal age (year)	31 (28, 32)	33 (29, 36)	0.396	0.079	31 (28, 32)	31 (29, 33)	0.038	0.879
Gravidity	1.9 (1.0, 2.0)	1.8 (1.0, 2.0)	0.041	0.848	1.8 (1.0, 2.0)	1.6 (1.0, 2.0)	0.144	0.561
Parity	1.0 (1.0, 1.0)	1.3 (1.0, 1.0)	0.576	0.018	1.0 (1.0, 1.0)	1.1 (1.0, 1.0)	0.144	0.562
Hypertensive disease in pregnancy	2 (5.9)	9 (16.1)	0.330	0.078	2 (6.1)	2 (6.1)	<0.001	0.606
Gestational diabetes mellitus	14 (41.2)	13 (23.2)	0.392	0.071	12 (36.4)	10 (30.3)	0.192	0.601
Cervical problems*	5 (14.7)	4 (7.1)	0.244	0.425	5 (15.2)	3 (9.1)	0.187	0.706
IVF-ET	20 (58.8)	27 (48.2)	0.254	0.329	18 (54.5)	20 (60.6)	0.108	0.618
MCDA twin	7 (14.7)	14 (25.0)	0.260	0.246	5 (15.2)	6 (18.2)	0.081	0.741
Gestational age at treatment (weeks)	29.0 (27.4, 31.0)	30.2 (28.2, 32.4)	0.450	0.041	29.0 (27.4, 30.1)	30.0 (28.2, 32.4)	0.324	0.192

Data presented as mean (25%, 75%) or N (%). Abbreviations: SMD, standardized mean difference; IVF-ET, in vitro fertilization-embryo transfer; MCDA, monochorionic diamniotic.

* Cervical problems encompassed cases of suspected cervical incompetence and patients who underwent a previous pregnancy with a loop electrosurgical excision procedure (LEEP).

### Maternal outcomes

**Fig 2:**
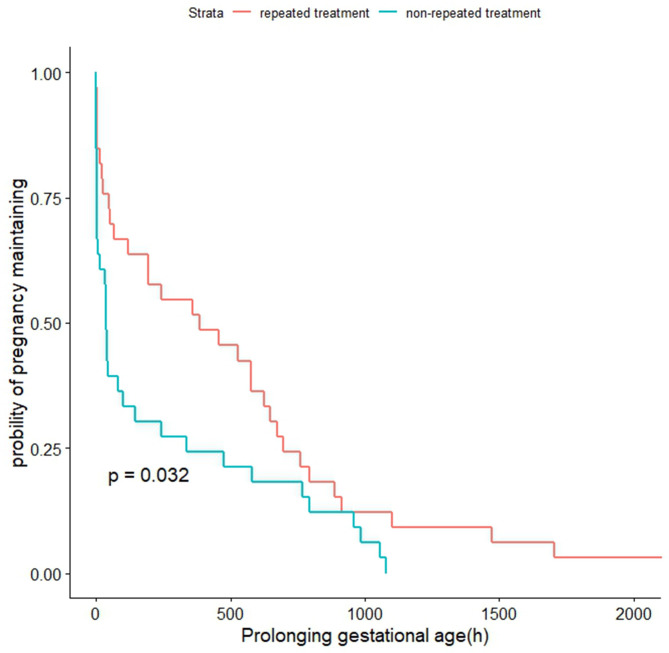
Kaplan-Meier curve that demonstrates the latency period (time from medication to delivery) in matched patients who either received continuous and repeated treatment of atosiban or did not. The survival curves depict the differences in delivery proportions between the two groups.

After matching, patients in the continuous and repeated treatment group had a longer duration of medication in the study (189.24 hours vs. 33.15 hours, p < 0.001), showed a significant latency period (683.77 hours vs. 272.32 hours, HR = 0.58 [0.35–0.96], p = 0.001), and had a later gestational age at birth (33.0 weeks vs. 31.5 weeks in the non-repeated treatment group, p = 0.042). Fig 2 illustrates that the latency period in the continuous and repeated treatment group was longer than that in the non-repeated treatment group (p = 0.032). There was a significant difference in the administration of antenatal steroids between the two groups (p = 0.002). A higher proportion of the continuous and repeated treatment group received a complete cycle of antenatal corticosteroids, while nine patients in the non-repeated treatment group had to discontinue antenatal steroid therapy due to clinical indications for delivery (seven cases of fetal distress and two cases of oligohydramnios). Another significant difference was observed in the type of labor (p = 0.010), particularly a higher rate of cesarean delivery in the continuous and repeated treatment group (93.9% vs. 63.7%). Further analysis of cesarean delivery revealed that a higher proportion of the non-repeated treatment group underwent the procedure due to fetal distress (33.3% vs. 6.5%, p = 0.004). No differences were found regarding other indications for the operation between the two groups. Regarding maternal outcomes, a lower percentage of patients in the continuous and repeated treatment group experienced postpartum hemorrhage (3.0% vs. 15.2%). No other significant differences were detected in maternal antenatal or postnatal outcomes. Additionally, there were no significant differences in patients diagnosed with chorioamnionitis between the two groups. One case of postnatal venous thromboembolism (VTE) was diagnosed in the continuous and repeated treatment group, while one case of antenatal sepsis and two cases of postpartum hypoxemia were diagnosed in the non-repeated treatment group. Both cases of hypoxemia involved mild postoperative pulmonary inflammation, which improved with supplemental oxygen and antibiotic therapy. Moreover, one case of puerperal infection was diagnosed in each group (Table 2).

**Table 2:** Maternal outcomes in matched population.

		Continuous and repeated treatment (n = 33)	Non-repeated treatment (n = 33)	*p* value	*HR* *(95% CI)*
Latency period (hour)		683.77 (144, 888)	272.32 (35, 384)	0.001	0.58(0.35,0.96)
		Continuous and repeated treatment (n = 33)	Non-repeated treatment (n = 33)	*p* value	*OR* *(95% CI)*
Duration of medication (hour)		189.24 (96, 192)	33.15 (15, 48)	<0.001	/
Gestational age at birth (weeks)		33.0 (32.3, 34.2)	31.5 (29.5, 33.3)	0.042	/
Cycle of antenatal steroids				0.002	/
Incomplete		0	9 (27.3)		
1 complete cycle		19 (57.6)	18 (54.5)		
2 complete cycles		14 (42.4)	6 (18.2)		
Delivery				0.010	/
Vaginal		2 (6.0)	11 (33.3)		
Operative vaginal		0	1 (3.0)		
Cesarean delivery		31 (94.0)	21 (63.7)		
Cesarean delivery analysis					
Elective operation		20 (64.5)	10 (47.6)	0.226	2(0.65,6.19)
Placenta previa		1 (3.2)	1 (4.8)	0.309	0.67(0.04,11.29)
Abnormal presentation		3 (9.7)	1 (4.8)	0.903	2.14(0.21,22.13)
Fetal distress		2 (6.5)	7 (33.3)	0.004	0.14(0.03,0.75)
Oligohydramnios		5 (16.1)	2 (9.5)	0.787	1.83(0.32,10.44)
Antenatal outcomes	*Chorioamnionitis*	10 (30.3)	4 (12.1)	0.130	3.15(0.87,11.36)
	*Sepsis*	0	1 (3.0)	1.000	/
Postnatal outcomes	*Postpartum hemorrhage*	1 (3.0)	5 (15.2)	0.199	0.18(0.02,1.59)
	*Postpartum hypoxemia*	0	2 (6.1)	0.492	/
	*Puerperal infection*	1 (3.0)	1 (3.0)	1.000	1(0.06,16.69)
	*VTE*	1 (3.0)	0	1.000	/

Data presented as mean (25%, 75%) or N (%). Abbreviations: VTE, venous thrombus embolism; CI, confidence interval.

### Neonatal outcomes

An apparent reduction in neonatal intensive care unit (NICU) admissions was observed in the continuous and repeated treatment group compared to the non-repeated treatment group (21.2% vs. 39.4%, OR=0.41 [0.19–0.89], p = 0.023). However, there was no significant difference between the two groups in terms of neonatal body weight or the incidence of neonatal adverse outcomes such as respiratory distress syndrome (RDS), acidosis, wet lung, sepsis, hypoglycemia, or feeding intolerance. With proper and effective treatment of the neonates, the length of stay in the NICU did not significantly differ between the two groups. Although there was a lower incidence of 1-minute, 5-minute, and 10-minute Apgar scores below 7 in the continuous and repeated treatment group, the difference was not statistically significant. Unfortunately, two twin babies born at 28 + 2 weeks of gestation died of severe RDS three days after birth (Table 3).

**Table 3:** Neonatal outcomes in matched population.

	Continuous and repeated treatment (n = 66)	Non-repeated treatment (n = 66)	*p* value	*OR* *(95% CI)*
Neonatal bodyweight (g)	1870.2 ± 489.9	1704.4 ± 515.7	0.061	/
1 min Apgar score <7	0	5 (7.6)	0.058	/
5 min Apgar score <7	0	2 (3.0)	0.496	/
10 min Apgar score <7	0	1 (1.5)	1.000	/
NICU admission	14 (21.2)	26 (39.4)	0.023	0.41(0.19,0.89)
Length of stay in NICU (day)	18.04 (14, 58)	22.55 (18, 60)	0.147	/
Respiratory distress syndrome	26 (39.4)	33 (50.0)	0.220	0.65(0.33,1.30)
Acidosis	40 (60.6)	40 (60.6)	1.000	1.00(0.50,2.01)
Wet lung	39 (59.1)	32 (48.5)	0.223	1.53(0.77,3.05)
Sepsis	0	2 (3.0)	0.496	/
Hypoglycemia	9 (13.6)	10 (15.2)	0.804	0.88(0.33,2.34)
Feeding intolerance	2 (3.0)	2 (3.0)	1.000	1.00(0.14,7.32)
Neonatal death	2 (3.0)	0	0.496	/

Data presented as mean±eD, or mean (25%,75%), or N (%). Abbreviations: NICU, neonatal intensive care unit; CI, confidence interval.

## Discussion

The aim of this study was to assess the effect and adverse outcomes of continuous and repeated treatment with atosiban in twin pregnancies. The main findings were as follows: (1) Women who received continuous and repeated treatment with atosiban experienced significant prolongation of pregnancy; (2) Neonates born after continuous and repeated treatment with atosiban had higher Apgar scores at birth, and the rate of NICU admission was reduced; and (3) There were no major antenatal or postnatal adverse outcomes for women who received continuous and repeated treatment.

The major strength of this study was that it was the first to investigate the continuous and repeated treatment of atosiban in twin pregnancies with threatened preterm labor. We collected five years of clinical data to evaluate the effectiveness and safety of continuous and repeated treatment with atosiban, which holds clinical significance in guiding practice. Additionally, in our study, we employed propensity score matching analysis, which effectively matched baseline characteristics between the two groups and had a similar effect to a randomized controlled trial in a retrospective study design. Nevertheless, the present study had some limitations. Firstly, despite collecting data over a period of five years, the sample size was still relatively small for a retrospective study conducted at a single center, which may introduce selection bias. Secondly, the assessment of cervical shortening was based on clinical examination by obstetricians and not on endovaginal ultrasonography screening, which could be considered for an updated clinical protocol in our hospital. Thirdly, cervical length data were not considered in PSM due to severe missing records in the original dataset. Lastly, a more rigorous comparison between cases completing 48-hour atosiban maintenance versus those receiving repeated courses remains warranted, which should be systematically addressed in our future study.

In this study, the mean duration of medication was 189.24 hours (equivalent to nearly 4 treatments of atosiban), and it effectively prolonged the latency period by 411.45 hours and the gestational age at birth by 1.5 weeks. Atosiban, an oxytocin receptor antagonist, was developed to delay preterm birth. In normal parturition, oxytocin stimulates contractions by converting phosphatidylinositol to inositol triphosphate, which binds to a protein in the sarcoplasmic reticulum, causing the release of calcium into the cytoplasm. Oxytocin receptor antagonists compete with oxytocin for binding to oxytocin receptors in the myometrium and decidua, thereby preventing the increase in intracellular free calcium that occurs when oxytocin binds to its receptor [24,25]. Although atosiban is not available in the United States, it is commonly used in Europe and other regions, including Shanghai, China. In our hospital, when a woman presents with threatened preterm labor, experienced obstetricians evaluate the situation and perform physical examinations to make a clinical decision regarding the administration of atosiban intravenously, taking into consideration factors such as cost and availability.

Gestational age is an important factor that affects neonatal prognosis [26]. Prolonging the gestational period can improve the outcome for the newborn without significantly increasing the risk to the pregnant woman. In our study, an increased gestational age was associated with greater neonatal body weight (an increase of 168g) and no neonates in the continuous and repeated treatment group had 1-minute, 5-minute, or 10-minute Apgar scores below 7. All neonates received a complete cycle of antenatal corticosteroid therapy before birth. After the infusion of atosiban ended, the plasma concentration of the drug rapidly decreased with a half-life of 0.21–1.7 hours. Therefore, the parent drug of atosiban is mainly eliminated metabolically by endopeptidase, resulting in few side effects on the fetus or neonates. There were no significant differences in short-term outcomes between the two groups, but there was a lower rate of NICU admission (a 17% decrease) in the continuous and repeated treatment group, and neonates were discharged from the NICU four days earlier. These findings suggest milder symptoms of complications after prolonged gestational age. Two neonatal deaths were attributed to very preterm labor and severe RDS.

The use of tocolytic therapy may provide short-term prolongation of pregnancy (up to 48 hours), allowing for the administration of antenatal corticosteroids and magnesium sulfate for neuroprotection, as well as facilitating transport to a tertiary facility [27]. The maintenance therapy with tocolytics for preventing preterm birth and improving neonatal outcomes remains controversial. In the past, long-term use of tocolytics was not recommended due to concerns about possible maternal side effects. However, continuous and repeated treatment with tocolytic therapy is necessary to prolong gestational age, alleviate family and social burdens, and help pregnant women build confidence in prolonging their pregnancies. Several clinical trials have focused on continuous and repeated treatment [5]. The commonly used regimen for nifedipine (immediate release) involves an initial oral dose of 20 mg, followed by 10 mg every 6 hours for 3–7 days or until transfer is completed [20]. However, there is still a lack of studies on the continuous and repeated treatment of tocolytic therapy for twin pregnancies. It is challenging for obstetricians to evaluate the effects of tocolytics and maternal complications or side effects simultaneously.

Multiple gestations are associated with a greater risk of maternal complications, including hypertensive disorders and metabolic problems [28]. Furthermore, they are more likely to be associated with maternal side effects, such as maternal tachycardia and pulmonary edema with beta2-agonists [13,14], and hypotension with calcium channel blockers [12], which may contribute to central nervous system disorders like headache and dyspnea. Atosiban is an optimized choice for twin pregnancies with threatened preterm labor due to its fewer maternal cardiovascular or pulmonary side effects. The overall frequency of side effects in patients receiving atosiban is significantly lower than that reported for any other drug used to inhibit preterm labor [17,29]. Therefore, it may be the treatment of choice for preterm labor, particularly in patients at risk of cardiovascular complications, including multiple pregnancies [30]. In our study, no maternal side effects (nausea, vomiting, flushing, headache, dizziness, or palpitations) were reported.

In the present study, we also assessed the antenatal and postnatal outcomes of pregnant women to consider the potential disadvantage of longer hospitalization. There were no significant differences in the rates of chorioamnionitis, sepsis during the antenatal period, or puerperal infection compared to the non-repeated treatment group. It is worth noting that multiple pregnancies are associated with an increased risk of venous thromboembolism (VTE) [31,32], and these pregnant women had to limit their activities due to inpatient intravenous medication and bed rest. In our study, there was one case of VTE in the continuous and repeated treatment group. Although the number is small, this finding underscores the need for proactive VTE prevention during pregnancy maintenance therapy. Additionally, two cases of hypoxemia occurred in the continuous and repeated treatment group, which may be related to prolonged bed rest during pregnancy. Therefore, moderate activity is encouraged during high-risk pregnancies to mitigate hypoxemia risks. However, with appropriate treatment, all patients recovered well and were discharged according to schedule. Another noteworthy finding in our study was that the incidence of postpartum hemorrhage did not increase with the duration of medication, suggesting that continuous and repeated treatment with atosiban did not affect uterine contractions postpartum.

## Conclusions

Continuous and repeated treatment with atosiban demonstrated significant efficacy in prolonging pregnancy for twin pregnancies at risk of preterm labor. Continuous and repeated treatment with atosiban was associated with lower fetal distress and lower NICU admission but no effect in adverse neonatal outcomes as well as the incidence of chorioamnionitis.
